# A Physical Activity Mobile Game for Hematopoietic Stem Cell Transplant Patients: App Design, Development, and Evaluation

**DOI:** 10.2196/20461

**Published:** 2021-04-13

**Authors:** Shannon Cerbas, Arpad Kelemen, Yulan Liang, Cecilia Sik-Lanyi, Barbara Van de Castle

**Affiliations:** 1 The Sidney Kimmel Comprehensive Cancer Center Johns Hopkins Hospital Baltimore, MD United States; 2 University of Maryland, Baltimore Baltimore, MD United States; 3 University of Pannonia Veszprém Hungary

**Keywords:** cancer, mobile app, gamification, bone marrow transplant, alpha testing, physical activity

## Abstract

**Background:**

Physical activity mobile apps may encourage patients with cancer to increase exercise uptake, consequently decreasing cancer-related fatigue. While many fitness apps are currently available for download, most are not suitable for patients with cancer due to the unique barriers these patients face, such as fatigue, pain, and nausea.

**Objective:**

The aim of this study is to design, develop, and perform alpha testing of a physical activity mobile health game for hematopoietic stem cell transplant (HSCT) patients. The ultimate future goal of this project is to motivate HSCT patients to increase physical activity and provide them with a safe and fun way to exercise.

**Methods:**

A mobile health game called Walking Warrior was designed as a puzzle game where tiles are moved and matched. Walking Warrior interfaces with an open-source step counter and communicates with a central online MySQL database to record game play and walking performance. The game came to fruition after following an iterative process model with several prototypes. Game developers and bone marrow transplant nurses were recruited to perform an expert usability evaluation of the Walking Warrior prototype by completing a heuristic questionnaire and providing qualitative suggestions for improvement. Experts also made qualitative recommendations for improvements on speed, movement of tiles, appearance, and accuracy of the step counter. We recruited 5 additional usability evaluators who searched for and compared 4 open-source step counter programs, then qualitatively compared them for accuracy, robustness, cheat proofing, ease of use, and battery drain issues. Patient recruitment is planned at a later stage in this project. This paper only describes software design, development, and evaluation, rather than behavioral evaluation (ie, impact on physical activity), which is the long-term goal of this project.

**Results:**

Internal consistency and the instrument’s reliability evaluation results from 1 clinical expert and 4 technical experts were deemed excellent (Cronbach α=.933). A hierarchical cluster analysis of the questionnaire item responses for similarity/dissimilarity among the experts indicated that the two expert groups were not clustered into two separate groups in the dendrogram. This indicates that the item responses were not affected by profession. Factor analyses indicate that responses from the 40-item questionnaire were classified into five primary factors. The associated descriptive statistics for each of these categories were as follows (on a scale of 1 to 5): clarity and ease (median 4; mean 3.7, SD 0.45), appropriateness (median 4; mean 3.7, SD 0.49), game quality (median 3.5; mean 3.3, SD 0.42), motivation to walk (median 3; mean 3.1, SD 0.58), and mental effort (median 3.5; mean 3.1, SD 1.27).

**Conclusions:**

The evaluation from experts and clinicians provided qualitative information to further improve game design and development. Findings from the expert usability evaluation suggest the game’s assets of clarity, ease of use, appropriateness, quality, motivation to walk, and mental effort were all favorable. This mobile game could ultimately help patients increase physical activity as an aid to recovery.

## Introduction

A hematopoietic stem cell transplant (HSCT) is the transplantation of stem cells, derived from bone marrow, peripheral blood, or umbilical cord blood, as a means of treatment for blood or bone marrow cancers. HSCT involves an intensive conditioning regimen that uses chemotherapy with or without total body irradiation; this is followed by a period of myelosuppression to create marrow space for the engraftment of the transplanted stem cells [1]. During the transplant process, many patients experience several physical and psychosocial complications and side effects, such as severe fatigue, loss of physical performance, infection, graft-versus-host disease, and distress [2]. Fatigue, a commonly reported symptom of patients who have undergone HSCT treatment, has multiple causes, including deconditioning, anemia, and medications. Regardless of the cause, fatigue impacts patients’ well-being, ability to reintegrate into their normal lifestyle, physical recovery from transplantation, and overall symptom management [1].

The Center for International Blood and Marrow Transplant Research showed an increase of 39% in allogenic transplants in individuals aged 60 years and older in the United States. In 2018, there were nearly 4000 transplants in the United States [3]. At the same time, smartphone use by patients with cancer is being utilized in many studies, suggesting that mobile health (mHealth) can be an effective means of patient engagement [4]. According to the National Comprehensive Cancer Network (NCCN), of all nonpharmacologic interventions, physical therapies and some psychosocial interventions have the strongest evidence base for treating fatigue in HSCT patients [5]. These interventions align with recommendations from the Oncology Nursing Society (ONS). The ONS presents a number of evidence-based interventions for cancer symptoms, which are published through critical reviews. Their review on fatigue confirmed exercise/physical activity to be an effective intervention in the management of cancer-related fatigue for patients with many types of cancer including HSCT [6]. Several meta-analyses have been conducted to provide a comprehensive evaluation of the impact of increased physical activity upon cancer-related fatigue [7-9]. This evidence was effective in understanding the need for mHealth for HSCT patients to help increase their physical activity levels.

The need to create a motivational game that would engage HSCT patients to be physically active is important for this population; they carry a smartphone (our survey in 2017 at the Johns Hopkins Bone Marrow Transplant unit found that >80% of the patients owned a smartphone), and the majority of patients receiving transplants are under 60 years of age [3]. Transplant patients also vary in their health care settings between inpatient and outpatient status. Having an app on their smartphones to use wherever they are for physical activity engagement is an appropriate solution. A systematic review by Hernandez Silva et al [10] showed that many mHealth interventions have potential benefits, and the most promising improvements are in fatigue outcomes. mHealth gaming can be used in patients with cancer and has the potential to improve treatment outcomes [10,11].

Exercise is not only safe during cancer treatment but can also improve physical function and quality of life [5]. Too much inactivity can lead to loss of body function, muscle weakness, and reduced range of motion. Regular exercise during cancer treatment can help lower the risk of falls, blood clots, nausea, and fatigue [12]. The NCCN Clinical Practice Guidelines for Cancer-Related Fatigue advise starting slowly with a 10-minute walk and incrementally progressing with distance and time [5]. The goal is to reach 30 minutes of aerobic exercise, 5 days per week [5]. Unfortunately, patients may find it difficult to reach the recommended levels of physical activity [13].

Smartphones are increasingly becoming integrated into our society and can serve as a tool to improve health outcomes. Kamboj and Krishna [14] illustrated the positive health impacts of an innovative smartphone gaming app, Pokémon GO (Niantic Inc), in which users encounter Pokémon monster avatars when walking around as opposed to traditional stationary/seated games [14].

The study by Brassil et al [15] included hospitalized HSCT patients in a trial of an incentive-based mobility program to maintain or improve fatigue. Their findings suggest that participating in mobility programs may minimize fatigue [10,15,16]. These examples help to establish the concept of gamification [17]—the process of using “game design elements in non-game contexts”—in the application of achieving an incentive for ambulation [18].

The use of mobile device apps to promote fitness may be helpful in increasing physical activity levels [19,20]. While there are many fitness and physical activity apps currently available for download, most of them center on measuring and improving athletic performance. Content analyses of serious games for health is limited, but comparing these results to those of nongamified health apps has shown that physical activity serious game apps demonstrate higher levels of behaviour theory [21].

Such apps are generally not well suited for most patients with cancer because they fail to address unique barriers, such as fatigue, pain, and nausea, that hinder this group from carrying out the recommended levels of physical activity [14]. We are aware of no mobile apps that promote physical activity specifically for HSCT patients. Therefore, innovative efforts are needed to develop and evaluate a mobile app that increases physical activity in this population. Meanwhile, we intend to design and develop software that is generic and suitable for a wide population (including patients with other types of cancer and people experiencing fatigue) to walk a medically prescribed number of steps.

Our intention is to develop a game, Walking Warrior (WW), to motivate HSCT patients to walk. Our rationale is as follows: (1) a large portion of HSCT patients have reported enjoying match-3 puzzle games such as Candy Crush, which is similar to our game; (2) continued game play requires walking: if patients want to play more, they will need to walk; (3) patients are advised that walking is part of their therapy so playing the game reinforces this behavior; (4) walking will allow players to unlock additional levels and allows them to earn higher scores; (5) game playing and walking performance data are automatically collected and displayed on a website that allows for patient self-tracking and provider review; (6) the game is mentally challenging, and this provides entertainment, opportunities for logical thinking, the element of chance, and high replayability; (7) the tiles that are moved in the puzzle are displayed as cell types and medications that are relevant to HSTC patients’ condition and educates players, thereby enhancing their knowledge of the underlying biology and treatment they receive; (8) in addition to their automatically collected data, patients will participate in a survey that will serve as a tool for software evaluation and additional development, which shows that the individual patient’s experiences and opinions are valued and will be integrated into the next phase of software development.

## Methods

### Overview and Planning

In this work, ideation, design, development, an expert heuristic usability evaluation (alpha testing), bug fixes, and prototypes of WW were conducted for HSCT patients. The purpose of combining a step counter with the game is to make engagement in physical activity more motivating and enjoyable [22,23]. Participants need to carry their phones to use the step counter app that runs in the background. The game progresses when the user walks the required number of steps and beats the puzzles (levels). Computer game developers, bone marrow transplant nurses, and nursing informatics students were recruited to evaluate the usability of WW and the step counter. The evaluations provide information to further improve game design and development to better suit patient needs.

The design and development process of WW took on a multidisciplinary approach with continuous systematic evaluations. The study was led by a computer science professor of nursing informatics, a nursing informatics student who is an oncology nurse, and an oncology nurse educator. The study team held meetings and communicated with computer programmers, an oncology research committee, domain experts of oncology, and domain experts of game design. The entire development process was based on a design that focused on the intended users, and prototype testing was performed throughout the life cycle.

To identify user preference for game type, a questionnaire was given to 30 HSCT patients. Inclusion criteria included users who (1) are >18 years of age, (2) are not working in health care, (3) have received HSCT therapy in the past, and (4) are currently playing a computer or mobile app game. Questions on the type of mobile games enjoyed by respondents, why they enjoyed it, and why they continue to play were asked. The majority of participants (n=21, 70%) preferred to play puzzle games, with half (n=11, 37%) preferring a match-3 puzzle game such as Candy Crush. Users of Candy Crush are not limited to any specific demographics; the game is played by users of all age groups, belonging to all ethnicities and religions, and in all 7 continents [24]. Therefore, WW was designed as a match-3 game for the enjoyment of the HSCT adult population and the game design was inspired by Candy Crush, but its rules, winning conditions, graphics, scoring, and sound effects are significantly different.

WW’s main objective is to increase the physical activity level of HSCT patients, who are the intended users. Specifically, our short-term target population is HSCT patients who are >18 years old, received walking instructions from their clinician as part of their recovery from bone marrow transplant, and are willing to play a puzzle game using their own Android device. To achieve this, the game is designed for each level to be unlocked after the user walks a clinically designated number of steps. The game has a step counter that tracks the steps of the user as they walk and rewards them with a token that can be used to unlock levels in the game. The game screen includes 9×6 moveable tiles that are displayed as biological cells, including red blood cells, white blood cells, platelets, neutrophils, stem cells, and nerve cells. The game also includes bonus cells, magnesium and potassium pills, as well as bricks and concrete blocks for added variety and difficulty. Cells and pills are relevant to the patients’ conditions and treatment. Each level has a customized goal that the player must attain to beat the level.

The targeted HSCT patient behavior change is use of our mobile health game, WW, as opposed to other apps. Through game design, we intend to prompt and motivate users to have increased physical activity in comparison to no app use. This may promote better engagement in patients’ prescribed therapy and adoption of physical activity. In addition, by playing WW, they will automatically provide data about their walking and game-playing behaviors through WW’s integrated step counter and the online database, which collects, stores, and displays the data. The database is designed to collect and display data to players and clinicians to instigate changes in behavior and physical activity level, motivate users, and track progress. This also serves as proof of game play and walking achievement, which are important for goal setting, goal achievements, self-monitoring, and fast, automated objective feedback. In the future, various competitions will be open to users; their scores will be visible to all competitors, hence encouraging them to achieve high scores. This is often done in the gaming industry to generate significant interest in game play and social community building. Players may choose to release their scores for public view in the database with a push of a button. There is no personally identifiable information in the database; only usernames and performance data are stored.

### Mobile Game Development and Prototypes

**Figure 1:**
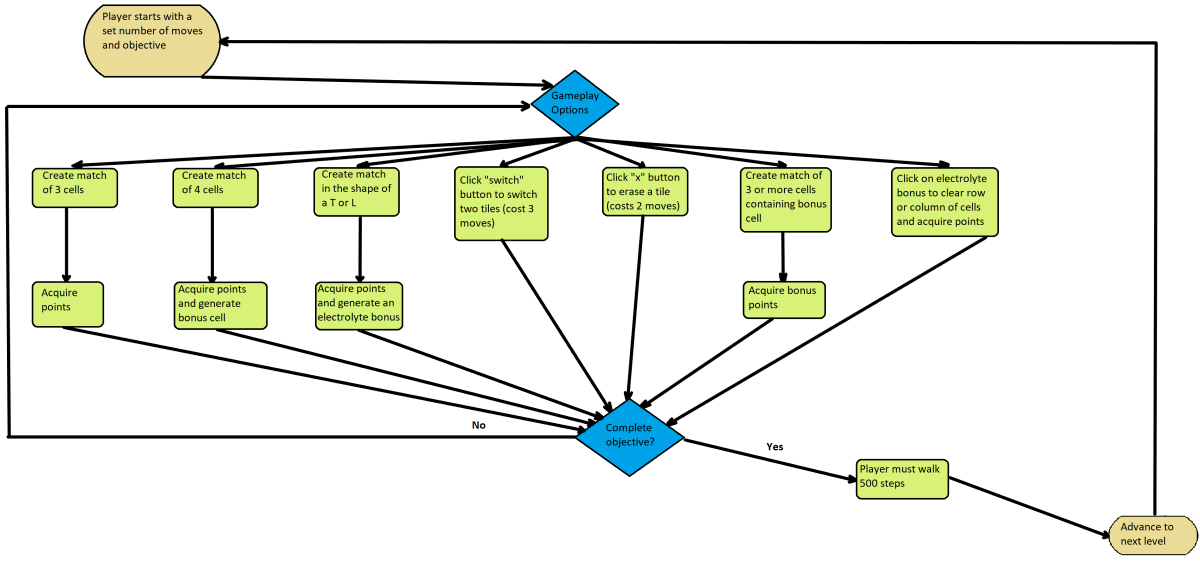
The game’s initial user interface design.

The game’s initial user interface (UI) design was sketched freehand and consisted of a login screen, menu page, cell art, tutorial content, and a game screen. Gameplay was then mapped out in Lucid Chart (Lucid Software Inc) and graphic art was designed in Paint.NET (dotPDN, LLC). The paper-based design was evaluated, refined, and adjusted based on team members’ feedback (Figure 1).

**Figure 2:**
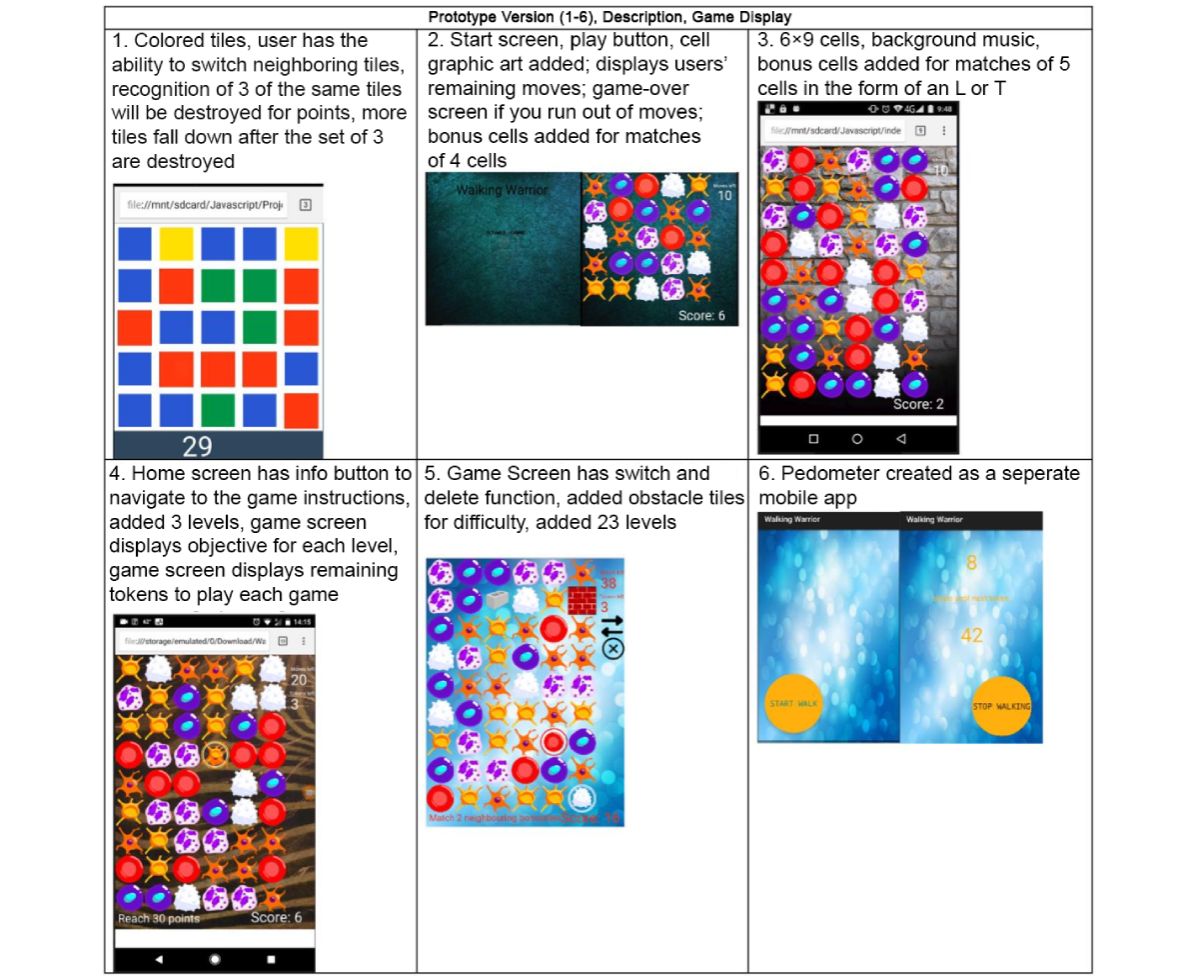
Prototype versions.

After several adjustments, the UI design was given to the programmers to be coded in JavaScript. A GitHub repository was created to store and share the source code for team members to view and test the game. WW functions on a web server, accessible through any device with an internet browser and an internet connection. Throughout development, the study team followed an iterative process model through a combination of design, testing, evaluation, and planning with each prototype version. During the testing process, team members navigated through the prototypes and reported bugs and recommendations for improvements. The iterative process ensured that with each new version, the identified problems would be fixed and requirements met (see Figure 2 for prototype versions).

**Figure 3:**
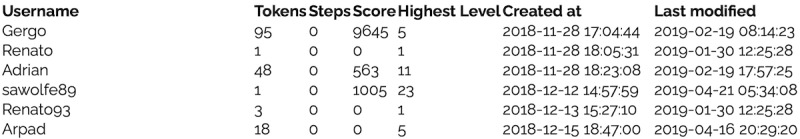
Screenshot of the MySQL database created to track patients’ behavior.

Separate JavaScript, Java, and PHP files were created for this game. The JavaScript files are responsible for the game itself. They manage the levels, contain game logic, and load the main frame and tiles. JavaScript is a lightweight, interpreted, object-oriented language with first-class functions and is best known as the scripting language for webpages. Java files count the steps and rely on the mobile device’s built-in accelerometers. Due to variations in mobile phone hardware and the limitations of open-source Java software, WW’s step counter currently only works on modern Android devices. The PHP files handle user logins and access and store the data on a server. The steps, login credentials, game-play performance, account creation, and the date and time of the last game played are stored in a MySQL database, as shown in Figure 3. The MySQL database is an open-source relational database management system. The database is stored on a server where administrators and authorized users can check the status of important variables for each user at any time. All data transfers go through AJAX, which does not require refreshing the webpage to send data through PHP, which makes the user experience smooth.

### Expert Usability Evaluation and Qualitative Data Collection

A 40-item expert heuristic questionnaire was designed to evaluate and assess the usability of WW. Two experts assessed the face validity of this questionnaire. A total of 9 questions were derived from the Perceived Health Website Usability Questionnaire (PHWSUQ), which is an existing validated tool [25]. The PHWSUQ consists of 12 items related to three subscales: (1) satisfaction, (2) ease of use, and (3) usefulness. The PHWSUQ has reported excellent reliability with a Cronbach alpha of .93. In our study, only 9 of the 12 items of the PHWSUQ were used because 3 items were not applicable to game evaluation. Moreover, 31 new questions were added to determine clarity and ease, appropriateness, game quality, motivation to walk, and mental effort (Multimedia Appendix 1). These questions were unique to the target user population and to the specific game we developed. The validation of the 31 questions we designed was done by 2 experts, of whom one was familiar with our topic and evaluated the questions to assess whether they successfully captured the topic. The second expert, who specialized in question construction, ensured that our survey did not contain common errors such as confusing or double-barreled questions.

Participants of the expert heuristic usability evaluation of WW included 4 game development experts and 1 bone marrow transplant nurse. Each participant played the game for a minimum of 2 hours in at least one session and randomly tested as many features as they could. Evaluators took notes and filled out a survey. They evaluated the step counter for functionality, robustness, and accuracy. They did not evaluate the step counter for its ability to motivate users or increase their physical activity level. This will be evaluated later with actual patients. The experts rated the questions on a scale of 1-5, where 1=strongly disagree, 2=disagree, 3=neutral, 4=agree, and 5=strongly agree. Participants also made comments and suggestions for improvement.

Step-counter software vary greatly due to the variations in hardware using different accelerometers, gyroscopes, GPS, sensitivity, and algorithms that classify smartphone moves into step events and nonstep events. This is in general a complex problem to investigate and address. We recruited 5 additional usability evaluators who were nursing informatics graduate students. They searched for and compared 4 open-source step-counter programs, then qualitatively compared them for accuracy, robustness, cheat proofing, ease of use, and battery drain issues. Generally, step counters lack perfection and have several usability and accuracy problems.

Behavioral evaluation (ie, impact on physical activity) will be done at a later stage of this project with HSCT patients.

### Data Analysis

The qualitative analysis was performed after the project’s nurse informaticist semantically merged, simplified, and summarized all the expert comments and requests into a list of nonredundant statements. These were discussed by the project team and given to the programmers for implementation.

Questionnaires were reviewed for reliability and validity of quality measures. A Cronbach alpha based on a 2-factor ANOVA (analysis of variance) was calculated for reliability, consistency, and reproducibility of the developed product. Descriptive statistics such as medians, means (SD), percentages of favorable and unfavorable ratings, and differential opinions between the bone marrow transplant nurse and computer game development experts were computed. Hierarchical cluster analysis and exploratory factor analysis were conducted to classify item responses for better interpretations. In addition, qualitative data from comments were summarized. All analyses were conducted using SAS (SAS Institute) and Microsoft Excel (Microsoft Corp).

## Results

We recruited 1 clinical domain expert and 4 game developers for an expert heuristic evaluation of our WW game prototype. Their ages ranged between 28 to 60 years and comprised 3 females and 2 males, with a master’s degree or higher. Analysis of the instrument demonstrated excellent internal consistency and reliability (Cronbach α=.933). Descriptive analysis showed that the overall game usability was favorable (>3) in all five categories, although two categories’ means were close to neutral (3.1). Table 1 provides the expert evaluations of 40 item responses and associated descriptive statistics, which showed some mean differences and agreements/disagreements between the two expert groups (the bone marrow transplant nurse and the game developers). However, a hierarchical cluster analysis of these item responses for similarity/dissimilarity among the experts indicated that the two groups were not clustered into two separate groups in the hierarchical cluster dendrogram. This indicates that the item responses were not affected by their profession if we consider the entire survey. Exploratory factor analysis indicate that 40 items were classified into five prime factors based on similarity, and means for each of the five categories were calculated to summarize item responses (Table 1).

Qualitative data suggest that the game is casually fun, suitable for the target audience, and the overall concept of the game has high potential. Experts recommended improvements on speed, ease of movement of tiles by finger, graphical quality of tile appearance, and accuracy of the step counter. They also recommended the addition of a “pause” and “back” button, and the addition of a tutorial for users unfamiliar with matching puzzle games.

After additional heuristic evaluation of the step counters done by the 5 nursing informatics students, based on the factors discussed in the *Methods* section, the old step counter was replaced and a new step counter was integrated into WW. A limitation of the selected step counter is that it only works on modern Android devices, since the open-source iPhone versions of step counters did not perform well. Developing our own step counter with better performance than the current best open-source step counter would be too complex, time consuming, and require extensive understanding and exploitation of different hardware technologies, artificial intelligence, machine learning algorithms, and tuning. Further, it would take several years of additional development and testing, followed by pairing this software with individual walkers to learn about and classify their steps based on their training data. Even then it would remain vulnerable to changing walking patterns among users in the future.

**Table 1:** Heuristic questionnaire results (n=5; 1 bone marrow transplant nurse, 4 game developers/computer science technical experts).

Category^a^		Median	Mean (SD)	Nurse score–developer mean	Agree (%)	Disagree (%)
**Clarity and ease**			3.7			
	Easy to read	2	2.4 (1.5)	–0.5	20	80
	Easy to learn	5	4.2 (1.3)	–2.75	80	20
	Easy to use	5	4.2 (1.3)	–2.75	80	20
	Easy to navigate	5	4.6 (0.5)	–0.75	100	0
	I made the desired moves with ease	5	4 (1.4)	–2.5	60	20
	Clear results of my actions	4	4 (1.2)	–2.5	80	20
	Clear display	4	4 (1.0)	0	60	0
	Easy to understand how to play	5	5 (0.0)	0	100	0
	Clear winning and losing criteria	4	3.6 (1.1)	–2	60	20
	Understood how steps convert into tokens	4	3.2 (1.1)	–1.5	60	40
	Recognized cells of the body	4	3.6 (1.1)	0.5	60	20
	Recognized magnesium and potassium pills	3	3 (1.6)	–1.25	40	40
	No problem accessing the step counter	2	2.8 (1.6)	–1	40	60
**Appropriateness**			3.7			
	Appropriate flow	4	4.2 (0.8)	–0.25	80	0
	Appropriate rules	4	3.8 (1.3)	–2.25	60	20
	Appropriate winning and losing criteria	4	4.2 (0.4)	–0.25	100	0
	Scores were assigned appropriately	4	4 (1.2)	–2.5	80	20
	Appropriate amount of time to win a level	4	3.8 (1.3)	–2.25	60	20
	Difficulty level appropriate for target patients	4	4 (1.0)	0	60	0
	Increase in difficulty was appropriate	4	3.6 (1.1)	–2	60	20
	Combos made the game more interesting	4	3.6 (1.1)	–0.75	60	20
	The game was free of bugs and problems	3	3.2 (1.3)	–1.5	40	40
	The step counter counted steps accurately	2	2.6 (1.5)	0.5	20	60
**Game quality**			3.3			
	Good appearance	4	3.8 (1.1)	0.25	80	20
	Good graphics	4	3.6 (1.1)	0.5	60	20
	Graphics added life to the game	4	4 (1.0)	0	60	0
	Pleasant music	3	2.8 (1.8)	0.25	40	40
	The game was entertaining	3	3 (1.0)	–1.25	40	40
	Had sense of immersion	3	3 (0.7)	1.25	20	20
	Provided sensory curiosity	3	3 (0.0)	0	0	0
	Felt satisfaction when beating levels	4	3.8 (1.6)	0.25	80	20
	Found the game to be highly replayable	4	3.2 (1.3)	1	60	20
	Found the game to be potentially competitive	3	3.2 (1.5)	–0.25	40	20
**Motivation to walk**			3.1			
	The game encouraged me to walk	3	2.6 (1.5)	–2	40	40
	This game will help me walk more	3	2.6 (1.5)	–2	40	40
	Desire to reach the next level motivated me to walk	3	3.2 (1.5)	1	40	20
	The game made walking more fun	4	3.2 (1.6)	1	60	40
	The game will motivate patients	4	4 (1.0)	1.25	60	0
**Mental effort (“disagree” answers are desired)**			3.1			
	Required too much mental effort	2	2.2 (1.3)	2.25	20	60
	Required too little mental effort	5	4 (1.7)	–3.75	80	20

^a^The heuristic questionnaire was organized into categories so statistical analysis could be calculated.

## Discussion

Our findings from the expert heuristic questionnaire suggest that WW’s clarity, ease of use, appropriateness, quality, motivation, and mental effort were moderately favorable. Experts offered many suggestions and recommendations that we used to improve the usability of the game. These resulted in bug fixes, modifications, and feature additions too numerous to individually mention here.

Although 2 experts assessed the face validity of the 40-item expert heuristic questionnaire we designed and used, it is not a measure with established psychometric properties. This is a limitation of our study. Nevertheless, an expert heuristic usability evaluation of games is an essential step in development. It is usually done as part of alpha testing before a game is given to the intended users for beta testing due to the large number of bugs and usability problems at this stage of development. It helps to significantly improve game quality without needing to recruit a large group of users who are not on the team. For complex games, this step is repeated many times by a small group of experts. Experts who understand both the subject domain and the game development process can identify most usability problems without prematurely recruiting a large sample of the intended users to confirm the bugs and usability problems the development team is already aware of. Recruiting intended users for usability evaluation is usually done during beta testing and/or after the game is given a “version 1.0” label, that is, when the game is no longer called a prototype but is referred to as a product. Development, however, often continues beyond version 1.0, and we plan to do so for WW as well based on data we receive from our intended users.

It is important to include experts from both domain expert backgrounds. Another limitation of this study is that we were only able to recruit 1 bone marrow transplant nurse to complete the expert heuristic usability evaluation of WW. Our research team included 2 additional bone marrow transplant nurses who participated in the software design but were not included in the expert heuristic evaluation to avoid potential biases in response.

After the above discussed expert heuristic usability evaluations, we expanded the testing team to include 30 graduate students in nursing informatics and in computer science at 3 universities. Various other volunteer testers were also recruited. A standard online Google Docs form was created to report bugs. Bugs can be reported by the push of a button in the game and are reviewed by the project leader and the programmers, and changes in the source code are made. Once the programmers have completed all known bug fixes and usability improvements, and fulfilled expert recommendations, we will perform a usability test with the target HSCT patient population at the Johns Hopkins Bone Marrow Transplant unit. The planned future human subjects protocol of this research has been approved by the Johns Hopkins Medicine Convened Institutional Review Board (IRB) and the University of Maryland, Baltimore IRB expedited review. Patients will be recruited, and informed consent will be obtained by study team members. No personal identifiable information will be collected for this study.

Future work will focus on evaluating suitability for the HSCT population. This will allow us to recruit adult bone marrow transplant patients to test the usability of the game using the System Usability Scale and a semistructured interview [26]. By determining the usability and user preferences of WW from HSCT patients, it will show us how to improve the game to better meet the needs of this patient population. Our ultimate goal is to increase patient awareness of the importance of physical activity and its effect on decreasing fatigue. If WW decreases fatigue by increasing the steps that patients walk, it may improve quality of life [12]. This game could ultimately help any patient needing to increase physical activity as an aid to recovery or even initiate a healthier lifestyle or serve as a form of entertainment. After HSCT patients pilot WW, we will adjust the game per their feedback and recommendations, and plan a rigorous evaluation that includes feasibility, acceptability, patient walking behavior, and measured impact on walking. Upon completion of these steps, we will consider releasing the game to the public as a therapeutic tool.

While our target population is HSCT patients, we have attempted to make the game generic enough for the wider public, which can be done by changing the graphics and the frequency and amount of steps needed to walk, which will allow individual users to set goals themselves rather than their clinicians. Ultimately, this mobile game with its associated step counter and database could help patients increase physical activity as an aid to recovery, which we expect to confirm in a quantitative way to support our goal in demonstrating their direct relationship.

## Appendix

Multimedia Appendix 1 Heuristic questionnaire.

## Abbreviations

ANOVA: analysis of variance
HSCT: hematopoietic stem cell transplant
IRB: institutional review board
mHealth: mobile health
NCCN: National Comprehensive Cancer Network
ONS: Oncology Nursing Society
PHWSUQ: Perceived Health Website Usability Questionnaire
UI: user interface
WW: Walking Warrior
