# Supplementary material for: A Physical Activity Mobile Game for Hematopoietic Stem Cell Transplant Patients: App Design, Development, and Evaluation
Source: JMIRx Med. 2021 Apr 13;2(2):e20461. doi: 10.2196/20461 (PMC10414428; doi:10.2196/20461)
Supplement: Multimedia Appendix 1 [file xmed_v2i2e20461_app1.docx]

| **Supplementary Materials**  Appendix Table: Heuristics Questionnaire: Rate on a Scale of 1-5 | |  |
| --- | --- | --- |
| I found it easy to read the information given in the game | 1 | |
| I found the game easy to learn | 2 | |
| I found the game easy to use | 3 | |
| The game was easy to navigate | 4 | |
| I was able to use my finger to make the desired moves with ease | 5 | |
| The results of my actions were clear | 6 | |
| The display with score, tokens, and game objective were clear | 7 | |
| The game allows users with no training to easily understand how to play | 8 | |
| The winning and losing criteria were clear | 9 | |
| I understood how the steps convert into tokens | 10 | |
| I recognized the cells in the game as cells in the body | 11 | |
| I recognized the pills in the game as magnesium and potassium | 12 | |
| I had no problem accessing the step counter and game on my phone | 13 | |
| The flow of the game was appropriate | 14 | |
| Game playing rules were appropriate | 15 | |
| The winning and losing criteria were appropriate | 16 | |
| The scores were assigned appropriately | 17 | |
| The amount of time needed to win a level was typically appropriate | 18 | |
| I believe that the difficulty levels are appropriate for the targeted patient population | 19 | |
| I believe that the amount of increase in difficulty levels was appropriate | 20 | |
| The cell combination bonuses made the game more interesting | 21 | |
| The game was free of bugs and problems | 22 | |
| I believe that the step counter counted my steps accurately | 23 | |
| The overall appearance of the game was good | 24 | |
| The overall quality of the graphics was good | 25 | |
| The graphics added life to the game | 26 | |
| The music in the game was a pleasant feature | 27 | |
| I found the game entertaining | 28 | |
| I had a sense of immersion in the game | 29 | |
| The game provided a sensory curiosity | 30 | |
| I felt satisfaction by beating the levels | 31 | |
| I found this game highly replayable | 32 | |
| I found this game potentially competitive through its scoring system | 33 | |
| The game encouraged me to walk more steps than I typically would | 34 | |
| Using this game will help me maintain better health habits by encouraging me to walk more | 35 | |
| The desire to reach the next level in the game motivated me to walk more | 36 | |
| The game made walking more fun | 37 | |
| This game would be helpful for someone going through bone marrow transplant treatment as motivation to walk every day | 38 | |
| Using this game required too much mental effort | 39 | |
| Using this game required too little mental effort | 40 | |
